# Bcl-3 regulates T cell function through energy metabolism

**DOI:** 10.1186/s12865-023-00570-3

**Published:** 2023-10-04

**Authors:** Hui Liu, Lin Zeng, Mengmeng Pan, Liwenhui Huang, Hanying Li, Mengxia Liu, Xinqing Niu, Chenguang Zhang, Hui Wang

**Affiliations:** Henan Key Laboratory of Immunology and Targeted Drug, Henan Collaborative Innovation Center of Molecular Diagnosis and Laboratory Medicine, School of Laboratory Medicine, Xinxiang Medical University, Xinxiang, China

**Keywords:** Bcl-3, Metabolism, Jurkat, T cells

## Abstract

**Background:**

Bcl-3 is a member of the IκB protein family and an essential modulator of NF-κB activity. It is well established that Bcl-3 is critical for the normal development, survival and differentiation of adaptive immune cells, especially T cells. However, the regulation of immune cell function by Bcl-3 through metabolic pathways has rarely been studied.

**Results:**

In this study, we explored the role of Bcl-3 in the metabolism and function of T cells via the mTOR pathway. We verified that the proliferation of Bcl-3-deficient Jurkat T cells was inhibited, but their activation was promoted, and Bcl-3 depletion regulated cellular energy metabolism by reducing intracellular ATP and ROS production levels and mitochondrial membrane potential. Bcl-3 also regulates cellular energy metabolism in naive CD4^+^ T cells. In addition, the knockout of Bcl-3 altered the expression of mTOR, Akt, and Raptor, which are metabolism-related genes, in Jurkat cells.

**Conclusions:**

This finding indicates that Bcl-3 may mediate the energy metabolism of T cells through the mTOR pathway, thereby affecting their function. Overall, we provide novel insights into the regulatory role of Bcl-3 in T-cell energy metabolism for the prevention and treatment of immune diseases.

**Supplementary Information:**

The online version contains supplementary material available at 10.1186/s12865-023-00570-3.

## Background

CD4^+^ T cells constitutively express TCR αβ and CD4 molecules, representing an integral element of the adaptive immune response with different immune functions [1, 2]. CD4^+^ T cells at different stages of development have different metabolic characteristics that are compatible with their immune function [3]. Naïve CD4^+^ T cells require relatively little energy to maintain essential functions, such as iron ion transport, the functional integrity of cell membranes, and protein structural conversion through the citric acid cycle and oxidative phosphorylation capacity [4]. The maintenance of energy metabolism in naïve CD4^+^ T cells requires the involvement of several signaling pathways, including interleukin 7 (IL-7)/interleukin 7 receptor (IL-7R) signaling, KLF2 (Kruppel-like Factor 2) and T-cell receptor (TCR) signaling [5]. Naïve CD4^+^ T cells are activated by antigenic stimuli and microenvironmental cytokines. To adapt to the various demands of cell growth, proliferation, differentiation and function, energy metabolism is transformed, and activated CD4^+^ T cells obtain energy mainly through aerobic glycolysis and glutamine cleavage pathways [3]. Current evidence suggests that alterations in metabolic pathways can regulate the function and differentiation of CD4^+^ T cells [6]. It was also found that various transcription factors and signaling pathways (mTOR, c-Myc, HIF1α, AMPK, etc.) participate in the metabolic regulation of activated CD4^+^ T cells, thus affecting the immune function of CD4^+^ T cells [4]. However, how metabolism-related molecules regulate the function of CD4^+^ T cells remains to be further investigated.

The B-cell lymphoma/lymphoma 3 (Bcl-3) gene is a subset of the nonclassical nuclear factor-κB (NF-κB) family [7, 8]. An increasing body of evidence suggests that Bcl-3 can selectively bind to the DNA-bound NF-κB p50 or p52 homodimer, regulating NF-κB-dependent gene transcription [9, 10]. In recent years, extensive research has shown that Bcl-3 is essential for the function of T cells [11–13]. Bcl-3 affects the survival of differentiated T cells and regulates T-cell proliferation as an environment-dependent modulator of T-cell responses [14]. Mice lacking Bcl-3 exhibited increased T-cell survival induced by adjuvant after exposure to superantigenic staphylococcal enterotoxin B (SEB) [15]. Bcl-3 overexpression slowed T-cell proliferation early in the process of the T-cell response to antigens both in vitro and in vivo [16]. Moreover, Bcl-3 plays an important role in the differentiation of T cells. The initial differentiation of both Th1 and Th17 cells has been reported to be normal in Bcl-3-deficient mice, and Bcl-3 inhibited the transformation of Th1 cells into Th17 cells by blocking the binding of c-Rel and p65 to NF-κB binding sites [17]. Bcl-3 can also promote Th2 cell differentiation through transcriptional activation of GATA3 [18]. In addition, conditional overexpression of Bcl-3 impaired the differentiation and development of Th1, Th2 and Th17 cells [19]. Altogether, the above findings suggest that the intrinsic expression level of Bcl-3 under steady-state conditions determines the fate of T cells.

The latest evidence suggests that Bcl-3 may serve as a novel metabolic modulator that governs lipid metabolism in obesity [20]. It has also been found that Bcl-3 mediates immune cell function by metabolism. The regulatory role of Bcl-3 on the antigen-presenting function of dendritic cells in adaptive immunity may be associated with the major transformation of metabolic programming [21, 22]. This finding suggests that Bcl-3 regulates T cell function through metabolic pathways. Therefore, we speculate that Bcl-3 is required to accurately control the function of immune cells in the dual context of metabolic changes and stress conditions to ensure an effective adaptive immune response. However, whether Bcl-3 modulates the function of T cells through metabolic pathways remains to be investigated.

In this study, the action of Bcl-3 on the proliferation and activation functions of T cells was further confirmed by knocking down Bcl-3 in Jurkat cells and naive CD4^+^ T cells, which inhibits the established regulatory effect of Bcl-3 on T-cell function. This finding suggests that the modulation of Bcl-3 on immune cells may be bidirectional. In addition, we provided hitherto undocumented evidence that Bcl-3 moderates the function of T cells by regulating their metabolic activity. The knockout of Bcl-3 affects the expression of metabolism-related genes in Jurkat cells, and the underlying mechanism may involve the mTOR pathway, warranting further investigation.

## Methods

### Reagents

Purified anti-mouse CD28 mAb, anti-mouse CD3 mAb, FITC-conjugated anti-mouse CD4 and PE-conjugated anti-mouse CD69 were purchased from BioLegend (San Diego, CA, USA). The Seahorse XF Glycolysis Stress Test Kit and Mitochondrial Stress Test Kit were provided by Agilent Technologies (Palo Alto, Calif.). Roswell Park Memorial Institute (RPMI) 1640, fetal bovine serum (FBS) and penicillin/streptomycin were ordered from Gibco (South Logan, UT, USA). DAPI (4′,6-diamidino-2-phenylindole) and 3-(4,5-dimethyl-2-thiazolyl)-2,5-diphenyl-2 H-tetrazolium bromide (MTT) were purchased from Solarbio (Beijing, China). Enhanced ATP Assay Kit, Mitochondrial Membrane Potential Detection Kit and Reactive Oxygen Detection Kit were supplied by Beyotime Biotechnology (Shanghai, China).

### Animals

The Bcl3^−/−^ mice derived from C57/B6N mice were constructed using the CRISPR/Cas9 system and gifted by Prof. Yinming Liang (Xinxiang Medical University). All mice were kept in specific pathogen-free conditions in an animal resource center at Xinxiang Medical University. All experiments were performed using female mice aged 6–8 weeks and in agreement with the animal care guidelines established by Xinxiang Medical University.

### Metabolic assessments

OCR/ECAR analysis was performed on the XFe24 Extracellular Flux Analyzer (Agilent). The seahorse instruments were preheated. One day before the assay, 1 mL XF Calibrant buffer was injected into the wells of a 24-well utility plate from Seahorse Bioscience and incubated at 37 °C overnight in a non-CO2 incubator. Seahorse XF base medium was used to prepare the test solution and was preheated to 37 ℃ before the test. Cells were seeded into an XFe24 microplate at a density of 5 × 10^5^ cells/well. When the detection reagent of ECAR/OCR was filled into the relevant injection port of the sensor cartridges, the cell microplates were incubated at 37 °C in a non-CO2 incubator for 45–60 min. Finally, the cell culture microplate was loaded onto the instrument after calibration. Wave 2.4 software (Agilent) was used to analyze the data.

### RNA isolation and qPCR

Total RNA was isolated from Bcl-3^−/−^ Jurkat cells and normal Jurkat cells using TRIzol reagent. Briefly, 2000 ng of total RNA was reverse transcribed using RT Master Mix (Takara). Q-PCR analyses were performed using SYBR Green SuperMix in an Applied Biosystems 7500 System. After normalization to the expression of HPRT by the 2^−ΔΔCT^ procedure, the relative mRNA level was revealed as the fold change relative to untreated controls. The specific primers used were synthesized by Sangon Biotech. Target mRNA levels were normalized to HPRT levels. The PCR primer sequences were as follows:

For Bcl-3, 5’-AACCTGCCTACACCCCTATAC-3’ (forward).

and 5’-CACCACAGCAATATGGAGAGG-3’ (reverse).

and for mTOR, 5’- ACTCGCTTCTATGACCAACTGA-3’(forward).

and 5’-TTTCCATGACAACTGGGTCATTG-3’ (reverse).

and for Raptor, 5’-ACTGGAACCTACCTTTGGCTT-3’ (forward).

and 5’-ACTGTCTTCATCCGATCCTTCA-3’ (reverse).

and for HIF1α, 5’- ACTGGAACCTACCTTTGGCTT-3’ (forward).

and 5’- ACTGTCTTCATCCGATCCTTCA-3’ (reverse).

and for S6K1, 5’- TTTGAGCTACTTCGGGTACTTGG-3’ (forward).

and 5’- CGATGAAGGGATGCTTTACTTCC-3’ (reverse).

and for Akt, 5’- TCCTCCTCAAGAATGATGGCA-3’ (forward).

and 5’- GTGCGTTCGATGACAGTGGT-3’ (reverse).

and for 4EBP1, 5’- CTATGACCGGAAATTCCTGATGG-3’ (forward).

and 5’- CCCGCTTATCTTCTGGGCTA-3’ (reverse).

and for LDHA, 5’- TTGACCTACGTGGCTTGGAAG-3’ (forward).

and 5’- GGTAACGGAATCGGGCTGAAT-3’ (reverse).

and for GLUT1, 5’- TCTGGCATCAACGCTGTCTTC-3’ (forward).

and 5’- CGATACCGGAGCCAATGGT-3’ (reverse).

and for HK2, 5’- TGCCACCAGACTAAACTAGACG-3’ (forward).

and 5’- CCCGTGCCCACAATGAGAC-3’ (reverse).

and for PKM2, 5’- ATAACGCCTACATGGAAAAGTGT-3’ (forward).

and 5’- TAAGCCCATCATCCACGTAGA-3’ (reverse).

and for MYC 5’- GGCTCCTGGCAAAAGGTCA-3’ (forward).

and 5’- CTGCGTAGTTGTGCTGATGT-3’ (reverse).

and for SREBF1, 5’- ACAGTGACTTCCCTGGCCTAT-3’ (forward).

and 5’- GCATGGACGGGTACATCTTCAA-3’ (reverse).

### Western blot analysis

The protein from Bcl-3^−/−^ Jurkat cells and normal Jurkat cells for Western blotting was extracted to determine the protein expression level. Western blot assays were performed with primary Abs against SREBF1, mTOR, P-AMPK, P-Akt and P-Raptor. Cells were collected and lysed in ice-cold WB/IP lysis buffer (Beyotime, Shanghai, China), and protease and phosphatase inhibitors were added. Then, after the nuclear proteins were extracted, the cell lysates or immunoprecipitates were separated by SDS‒PAGE and transferred to PVDF membranes according to the manufacturer’s instructions. After blocking with 5% skim milk, the membranes were incubated with the primary antibody at 4 °C overnight and then with the corresponding secondary antibody for 1 h at room temperature. The membranes were washed with TBST buffer three times for 10 min each. Finally, the membrane was placed into the instrument for exposure, and pictures were taken.

### Determination of intracellular ATP levels

Jurkat and Bcl-3^−/−^ Jurkat cells were seeded into a 24-well culture plate at a density of 1.2 × 10^5^ cells/well, and each group was set up with 4 duplicate holes. After 24 h, the medium was discarded, and ATP lysate was added to each well to lyse the cells. Cells were centrifuged at 12,000 r/min for 5 min. The supernatant was collected from each of the wells and detected with an ATP detection kit. Finally, the ATP concentration of each well was calculated according to the standard curve.

### Measurement of ROS levels

A total of 1 × 10^6^ Jurkat and Bcl-3^−/−^ Jurkat cells were incubated in 24-well plates for 24 h to determine the level of reactive oxygen species (ROS). Subsequently, the cells were stained with DCFH-DA (10 µM) for 20 min and treated with CCCP (10 µM) as a positive control. Finally, after staining, the cells were washed and resuspended in PBS buffer and then analyzed by flow cytometry.

### Analysis of mitochondrial membrane potential

The Mitochondrial Assay Kit (Beyotime, Shanghai, China) was used to measure the mitochondrial membrane potential (ΔΨm). A total of 1 × 10^6^ Jurkat and Bcl-3^−/−^ Jurkat cells were added to 0.5 mL diluted 1×JC-1 staining working buffer and mixed well. The cells were incubated in a 37 ℃ incubator for 20 min and then collected by centrifugation. Next, the cells were washed twice with JC-1 (1×) dyeing buffer, and then the cells were resuspended in the dyeing buffer for analysis by flow cytometry. Moreover, the resuspended cells were fixed with an appropriate amount of paraformaldehyde fixing solution, and then the nuclei of the cells were stained with DAPI and visualized with confocal laser scanning microscopy (CLSM).

### Proliferative ability

Jurkat and Bcl-3^−/−^ Jurkat cells were collected and coincubated with 5 µM CFSE. Next, 5 × 10^5^ cells/ml was suspended in 1640 medium with 10% FBS, and then 1 ml/well cell suspension was added to the 24-well plates. After 3 days, the proliferative ability of the cells was determined by flow cytometry.

### Activation ability

Briefly, 5 × 10^5^ Jurkat or Bcl-3^−/−^ Jurkat cells were added to 48-well plates precoated with CD3 antibodies at 0.5 µg/ml per well. Then, 10 µg/ml anti-mouse CD28 antibody was added and incubated for 24 h. Then, the cells were stained with CD69 antibody, and the expression of CD69 was determined by flow cytometry.

### Detection of the apoptosis and IFN-γ production

5 × 10^5^ Jurkat or Bcl-3^−/−^ Jurkat cells were added to 48-well plates precoated with CD3 antibodies at 0.5 µg/ml per well. Then, 10 µg/ml anti-mouse CD28 antibody was added and incubated for 24 h. The cells were then collected and stained with annexin V and PI for 20 min at room temperature or stained with IFN-γ, then the cells were analyzed using flow cytometry.

### T-cell isolation and stimulation

Bcl-3^−/−^ and normal wild-type mice were euthanized by cervical dislocation quickly. Spleens from the mice were ground and filtered through 40 μm mesh to obtain a single-cell suspension. The red blood cells were subsequently depleted from the cell suspension with erythrocyte lysate. Following erythrocyte lysis, 5 × 10^5^ cells/well were inoculated into 48-well plates with a 500 µL system per well. After 24 h, the cells were collected for ECAR analysis or flow cytometry.

### Flow cytometry

Jurkat cells or single-cell suspensions from the spleen were washed and initially stained with CD4-FITC and CD69-PE. Then, the cells were stored at 4 °C in the dark for 30 min and washed with PBS 3 times. Activated T cells were identified by CD4^+^CD69^+^. CD4^+^ T cells were gated by CD4^+^. CD69^+^ T cells were gated by CD69^+^. We performed flow cytometry using a BD FACS Canto™ flow cytometer. FlowJoV10 was used to analyze flow data.

### Statistical analysis

We carried out statistical analysis using GraphPad Prism 8.0. All data on the graphs are presented as the mean ± SD. Two-tailed Student’s t test was used to analyze the differences among the study groups, and a P value < 0.05 was statistically significant.

## Results

### Bcl-3 depletion decreased the energy metabolism of jurkat cells

To evaluate the effects of Bcl-3 on the metabolism and function of CD4^+^ T cells, Jurkat-derived cell lines lacking the Bcl-3 gene (hereafter termed Bcl-3^−/−^ Jurkat cells) were used as effector cells, and normal Jurkat cells were used as control cells in our experiments. The subtypes of these two cell types were characterized by flow cytometry. Overwhelming evidence substantiates that both Jurkat cells and Bcl-3^−/−^ Jurkat cells belong to the subgroup of CD4^+^ T cells (data not shown), which provides a cell model for further research.

**Fig. 1:**
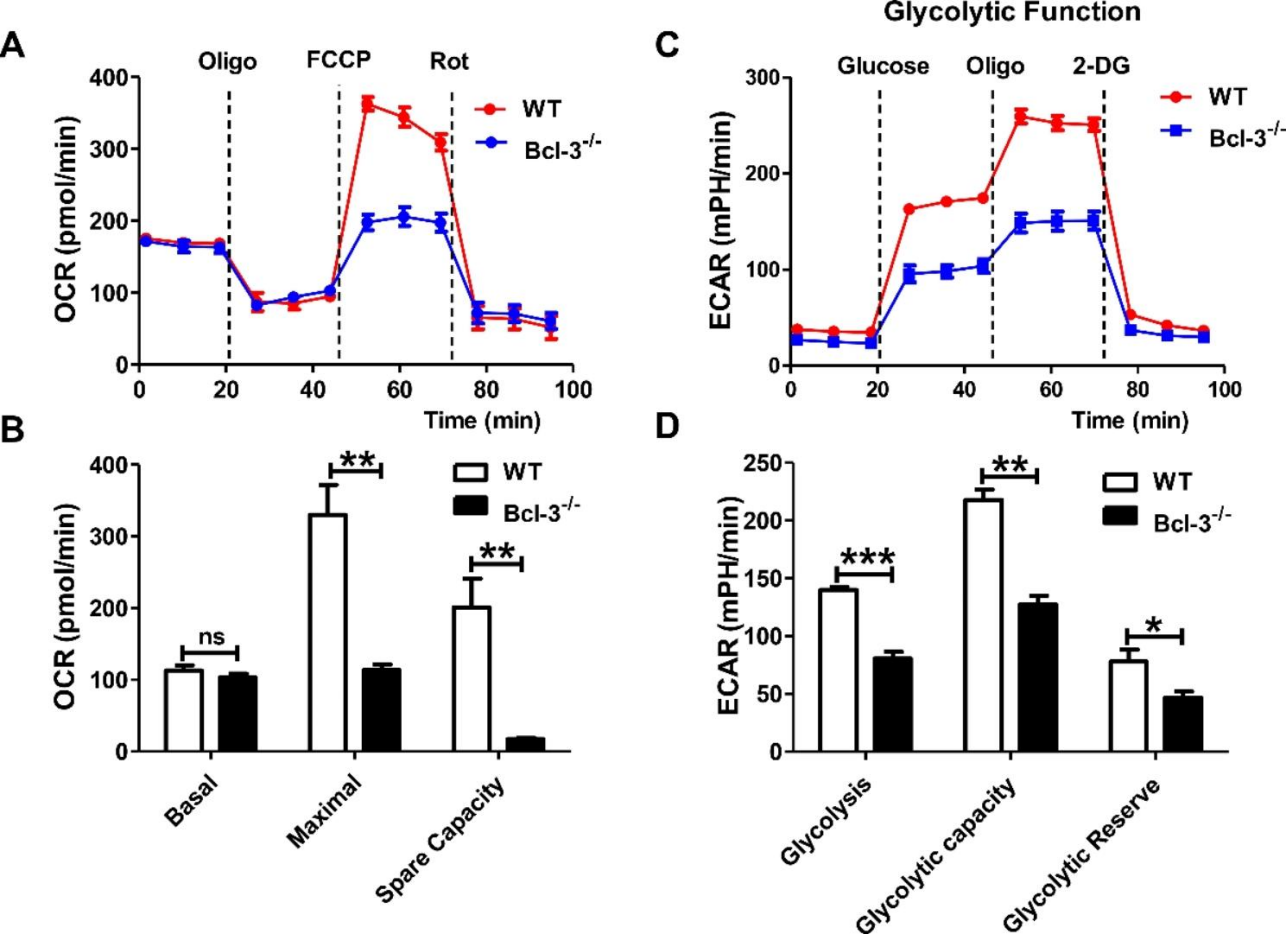
Bcl-3 depletion decreased the energy metabolism of Jurkat cells. Cells (5 × 10^5^) were seeded in 96-well XF Seahorse incubation plates as indicated in the protocol. Cells were cultured at 37 °C in XF base medium (pH 7.4), and FCCP (1 µM), oligomycin (1 µM), Rot (0.5 µM), glucose (10 mM) and 2-DG (50 mM) were added sequentially into the plates at specific time points following the manufacturer’s guidelines. The oxygen consumption rate (**A**, **B**) and extracellular acidification rate (**C**, **D**) of Jurkat and Bcl-3^−/−^ Jurkat cells were determined using an XF24e extracellular flux analyzer

To assess the metabolic effects driven by Bcl-3, we employed the XFe24 Extracellular Flux Analyzer (Seahorse Biosciences) to estimate the oxygen consumption rate (OCR) and extracellular acidification rate (ECAR) to quantify the levels of oxidative phosphorylation (OXPHOS) and glycolysis, respectively. The results showed that although the basal aerobic respiration rate of Bcl-3^−/−^ Jurkat cells did not significantly change, the ultimate respiration rate and reserve respiration capacity induced by carbonyl cyanide 4-(trifluoromethoxy) phenylhydrazone (FCCP) were remarkably lower than those of control cells (Fig. 1A, B). In addition, compared to control cells, Bcl-3 knockout cells exhibited markedly decreased glycolysis and glycolytic capacity (Fig. 1 C, D). The above results suggest that Bcl-3 is involved in mitochondrial energy metabolism and affects the energy metabolism mode of Jurkat cells.

### Bcl-3 depletion decreased the production of ATP and mitochondrial membrane potential and increased the level of ROS

**Fig. 2:**
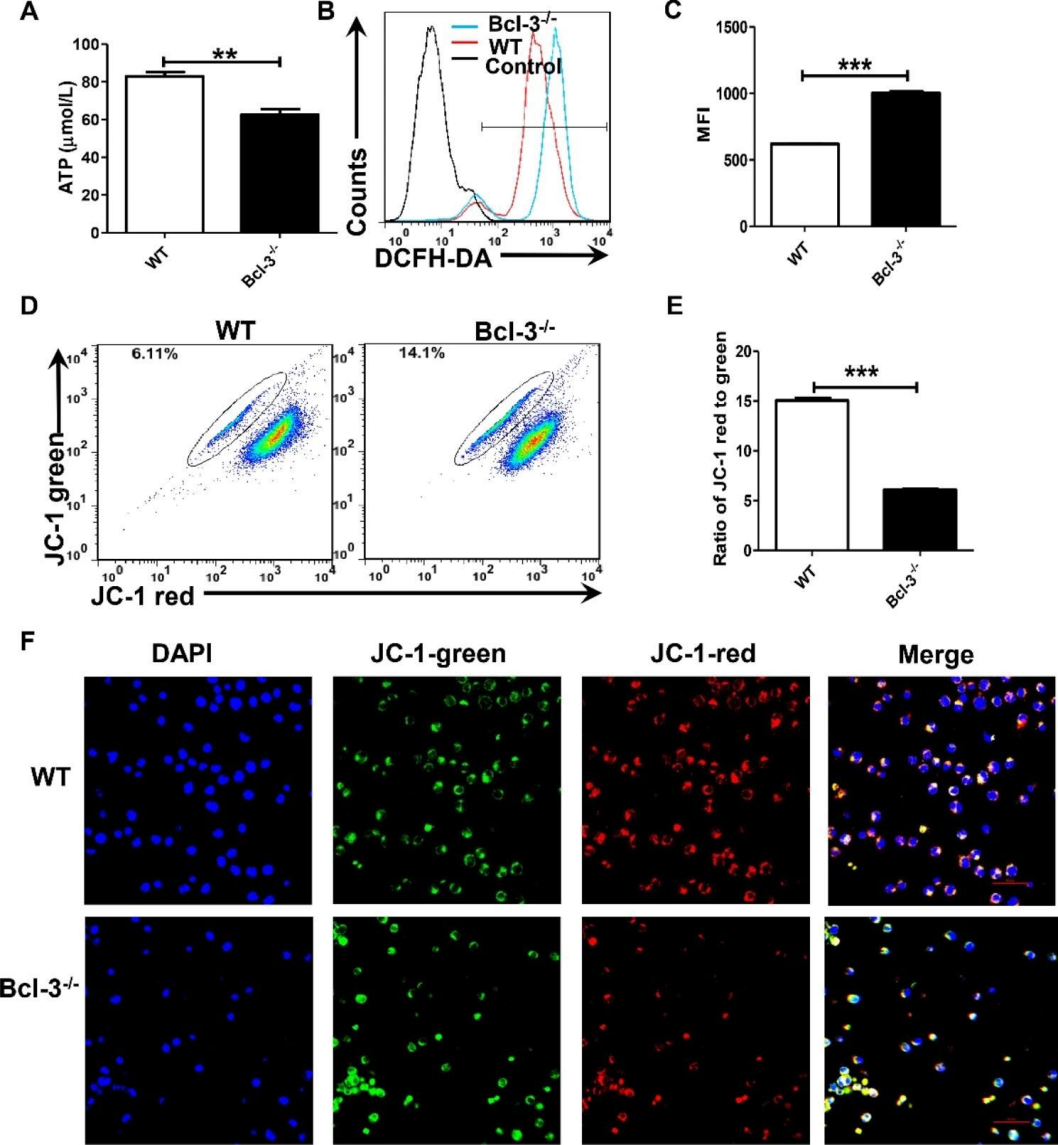
Bcl-3 depletion decreased the production of ATP and mitochondrial membrane potential and increased the level of ROS. (**A**) Jurkat and Bcl-3^−/−^ Jurkat cells were incubated for 24 h, and ATP levels were measured using the ATP assay kit. (**B**, **C**) The intracellular ROS levels were measured using the ROS assay kit. The mitochondrial membrane potential was determined by flow cytometry (**D**, **E**) and confocal microscopy (**F**)

Previous experiments implied that the knockout of the Bcl-3 gene decreased the energy metabolism of cells. Given the importance of the mitochondrial respiratory chain for energy metabolism and decreased OCR after Bcl-3 knockout, we assessed whether Bcl-3 could regulate the production of ATP and ROS and mitochondrial membrane potential, which are important to mitochondrial function. To confirm the involvement of Bcl-3 in ATP production, an ATP assay kit was used to measure ATP levels in Jurkat and Bcl-3^−/−^ Jurkat cells. Bcl-3 knockout decreased ATP production in Bcl-3^−/−^ Jurkat cells compared to control Jurkat cells (Fig. 2A). We also determined the levels of intracellular ROS in Jurkat and Bcl-3^−/−^ Jurkat cells by staining with a ROS-specific fluorescent probe followed by flow cytometry (Fig. 2B, C). Elevated intracellular reactive oxygen species (ROS) levels were observed in the Bcl-3 knockout cells. This finding suggests that Bcl-3 mediates ROS production and energy metabolism in Jurkat cells.

In addition, to verify the effect of Bcl-3 on mitochondrial respiration, JC-1 staining was performed to evaluate the mitochondrial membrane potential. Flow cytometry analysis showed that the green fluorescent signal, which represents a lower mitochondrial membrane potential, was significantly higher in Bcl-3^−/−^ Jurkat cells. This finding demonstrates that Bcl-3 deficiency could reduce the mitochondrial membrane potential of Bcl-3 knockout Jurkat cells compared to control Jurkat cells (Fig. 2D, E). The confocal microscopy results were consistent with the flow cytometry results (Fig. 2F). In summary, we found that Bcl-3 depletion can increase ROS accumulation, reduce the mitochondrial membrane potential of Jurkat cells, and inhibit the synthesis of intracellular ATP. Bcl-3 plays an essential role in the energy metabolism of Jurkat cells.

### Bcl-3 deficiency inhibits Jurkat cell proliferation but promotes Jurkat cell activation

**Fig. 3:**
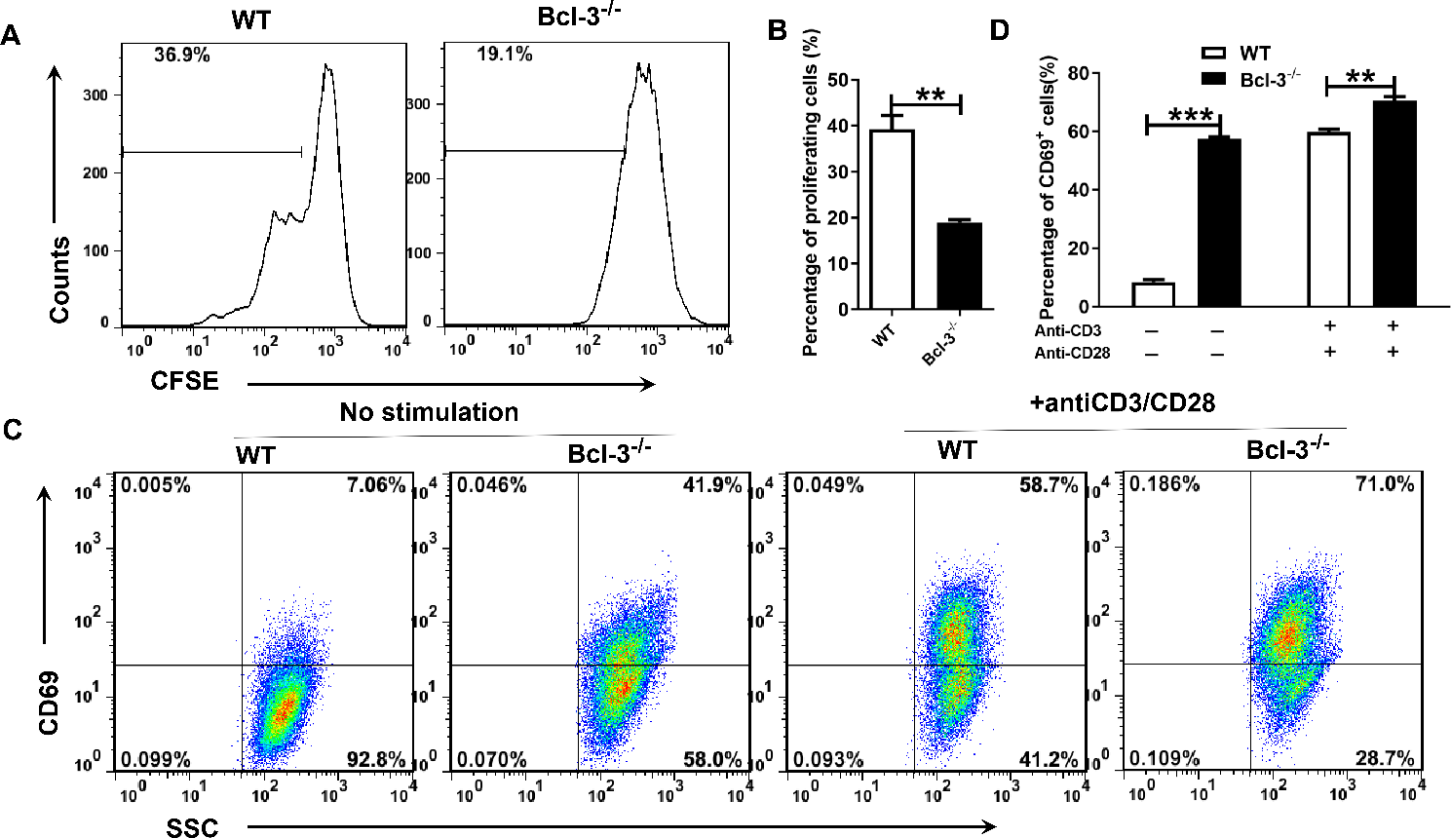
Bcl-3 deficiency inhibits Jurkat cell proliferation and promotes its activation. (**A**, **B**) Jurkat and Bcl-3^−/−^ Jurkat cells labeled with CFSE were cultured for 3 days, and then the proliferation of cells was determined by flow cytometry. (**C**, **D**) Cells were activated with or without anti-CD3/CD28. After 24 h, the cell surface was stained with an anti-CD69 antibody, and activation was determined by flow cytometry

**Fig. 4:**
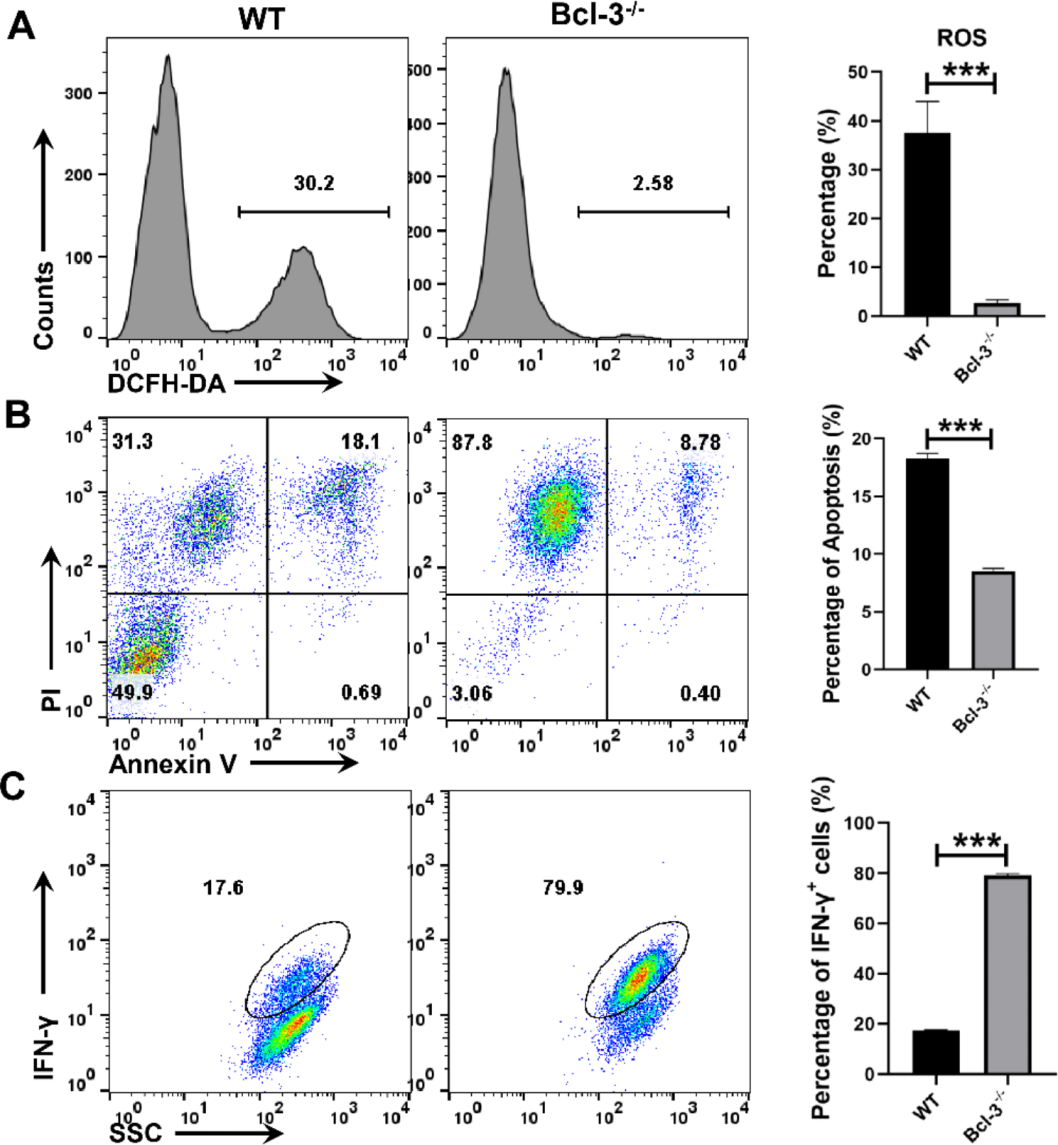
Bcl-3 deficiency inhibits ROS production and promote the necrosis and IFN-γ production of cells with stimulated by CD3/CD28. Jurkat and Bcl-3^−/−^ Jurkat cells were activated with anti-CD3/CD28 for 24 h. (**A**) The intracellular ROS levels were stained using the ROS assay kit and determined by flow cytometry. (**B**) Cell apoptosis (**B**) and IFN-γ production (**C**)were determined by flow cytometry

It is well established that there is a dynamic and precise relationship between metabolic regulation and the function of specific cell types that are involved in the immune response [23]. In this study, we labeled Jurkat and Bcl-3^−/−^ Jurkat cells with CFSE to characterize the influence of Bcl-3 on T-cell proliferation. The proliferation of cells was evaluated by flow cytometry. The proliferation rate of cells was lower in Bcl-3^−/−^ Jurkat cells than in normal Jurkat cells (Fig. 3A, B). We further analyzed the state of cell activation, and CD69 was used as a marker of activation. The results showed that the activation in Bcl-3^−/−^ Jurkat cells was significantly higher than that in control group cells, irrespective of activation with anti-CD3/CD28 (Fig. 3 C, D). Moreover, we found that the Bcl-3^−/−^ Jurkat cells activation with anti-CD3/CD28 produced decreased ROS and induced stronger necrotic than Jurkat cells(Fig. 4A, B). In addition, we also analyzed the cell effector functions by detecting the production of IFN-γ, the results suggested that Bcl-3^−/−^ Jurkat cells produced more IFN-γ than Jurkat cells when stimulated with anti-CD3/CD28(Fig. 4C). In conclusion, the Bcl-3 gene affects cell proliferation and activation and plays an essential role in the function of T cells.

### Bcl-3 regulates the expression of metabolism-related genes

**Fig. 5:**
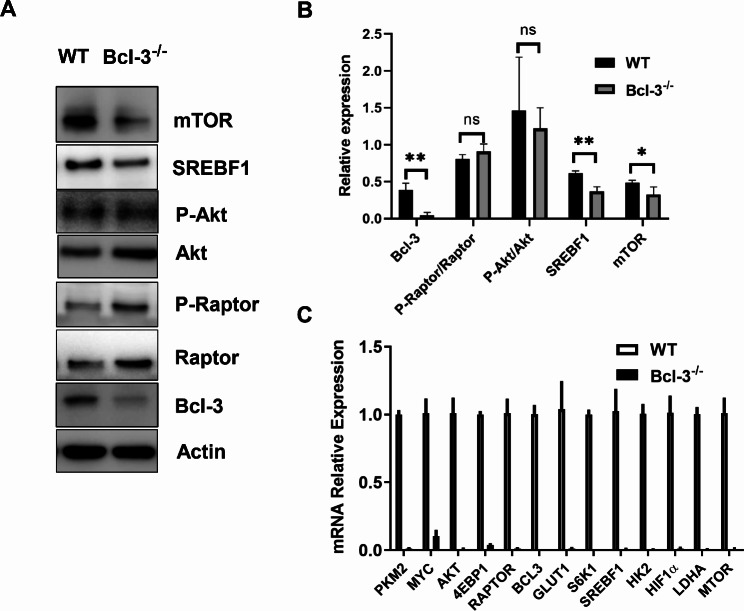
Bcl-3 regulates the expression of metabolism-related genes. (**A**) The expression of metabolism-related genes was detected by Western blot, cropped gels and blots are shown, and the full-length original blot or gel is included in the additional files. (**B**) The expression of metabolism-related genes was measured by RT‒PCR. One representative blot out of three is shown

Metabolism plays an integral role in many cellular, tissue, and body processes, while the immune response has a far-reaching impact on metabolism [24]. Based on the above results, we sought to investigate the underlying mechanisms by which Bcl-3 affects T-cell energy metabolism; we evaluated the key enzymes and transcription factors related to metabolism. Western blot indicated that Bcl-3 knockout significantly reduced the expression levels of SREBF1 and mTOR, whereas the level of phosphorylated Akt had no changes and Raptor expression levels increased (Fig. 5A, B). In addition, qPCR analysis indicated that Bcl-3 knockout markedly downregulated the expression of critical molecules in the PI3K/AKT/mTOR signaling pathway, such as AKT, mTOR, SREBF1, PKM2, S6K1, 4EBP1, GLUT1, HIF1α and HK2 (Fig. 5C). In summary, the Bcl-3 gene affects the expression of key enzymes and transcription factors related to metabolism, and the specific mechanisms may be involved in the mTOR signaling pathway.

### Bcl-3 deficiency regulates the metabolism of CD4^+^ T cells

**Fig. 6:**
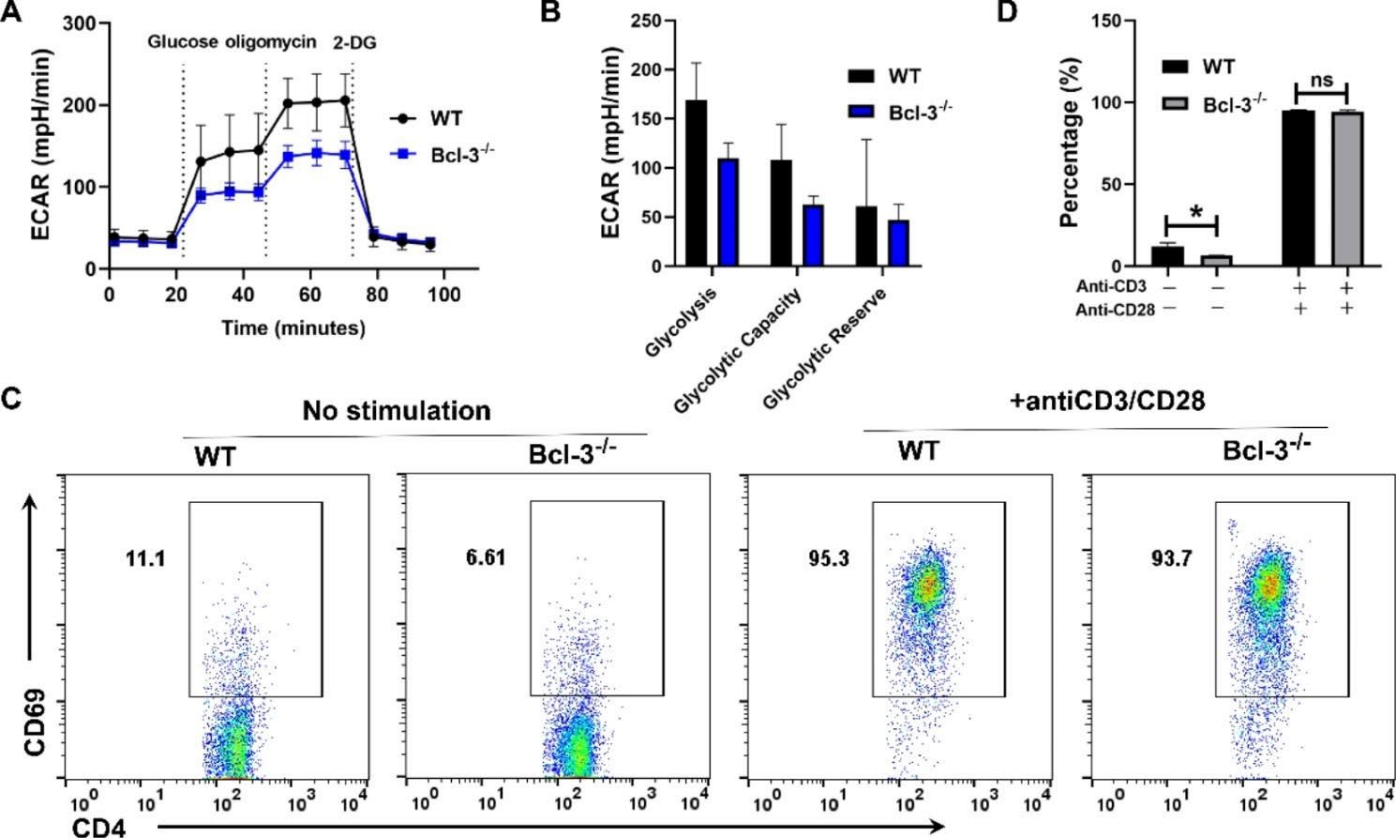
Bcl-3 deficiency regulates the metabolism of CD4^+^ T cells. The extracellular acidification rate of naïve CD4^+^ T cells isolated from the spleens of Bcl-3^−/−^ and WT mice was determined using an XF24e extracellular flux analyzer (**A**, **B**), and their activation was determined by flow cytometry (**C**, **D**) (n = 5)

To further corroborate the above conclusions at the level of primary cells, we isolated naïve CD4^+^ T cells from the spleens of Bcl-3-deficient (Bcl-3^−/−^) and normal wild-type mice and cultured them with anti-CD3/CD28 for 24 h. A Seahorse XFe24 analyzer was used to evaluate their metabolic indices. As shown in Fig. 6A, B, the extracellular acidification rate (ECAR) of naive T cells derived from Bcl-3^−/−^ mice was markedly reduced compared to that of T cells derived from wild-type mice. This result is consistent with the findings of the above cell lines and further corroborates that Bcl-3 affects the energy metabolism of T cells in vivo.

In addition, we evaluated the activation of these cells by flow cytometry. We found that the activation of CD4^+^ T cells derived from the spleen of Bcl-3^−/−^ mice was decreased compared with the activation of CD4^+^ T cells derived from the spleen of WT mice, while there was no significant difference in T-cell activation between the naive T cells derived from the spleen of Bcl-3^−/−^ mice and control mice, which were stimulated with anti-CD3/CD28 (Fig. 6C, D).

## Discussion

Over the years, the involvement of Bcl-3 in T-cell function has been gradually revealed. In this research, we substantiated the critical role of Bcl-3 in the proliferation and activation of T cells. In Jurkat cells, knockout of the Bcl-3 gene resulted in a reduction in the proliferation rate and a significant increase in the ratio of activated cells, consistent with previous findings of the effect of Bcl-3 on T-cell proliferation. T cells from mice lacking Bcl-3 exhibited limited proliferation in response to T-cell receptor (TCR) stimulation [25]. However, the effects of Bcl-3 on T-cell function appear to differ. In naive CD4^+^ T cells, we observed that Bcl-3 had no significant effect on activating CD4^+^ T cells after costimulation with CD3 and CD28, and there was a reduction in basal CD69 expression given that these cells were not activated by any stimulus. Bcl-3 has been reported to boost the survival of activated CD4^+^ T cells in response to adjuvant immunization [26]. Some researchers believe that the dual regulatory role of Bcl-3 on T-cell function is intrinsic to T cells specifically expressing Bcl-3 and does not require the assistance of antigen-presenting cells [16]. These experimental results suggest that Bcl-3 is an environment-dependent modulator of T-cell function, and the molecular mechanisms underpinning its function are still being revealed. In summary, Bcl-3 exhibits dual regulatory roles since it regulates biological immune function, and it is plausible that regulation of Bcl-3 expression is important for T-cell homeostasis and function, which warrants further studies.

In recent years, it has become increasingly apparent that the biological processes of organisms are linked to profound changes in metabolism and that cell fate and function are regulated by metabolism [27–29]. More importantly, the role of Bcl-3 in influencing biological processes by controlling metabolism has gradually emerged. Research suggests that Bcl-3 may be a potential new modulator of lipid metabolism in the development of obesity by establishing an obese mouse model under a high-fat diet [20]. The knockout of Bcl-3 significantly downregulated SREBP1 [20], an enzyme related to lipid anabolism, consistent with the significant downregulation of SREBP1 by Jurkat cells with Bcl-3 knockout in our present study. More importantly, our results showed that the extracellular acidification rate and glycolysis levels were significantly reduced in Jurkat cells with Bcl-3 knockout. Further studies showed that reduced energy metabolism was mainly caused by ROS accumulation, decreased mitochondrial membrane potential, and ATP synthesis inhibition. As the increased ROS content may influence the oxidative stress and promote the apoptosis and IFN-γ production of Jurkat cells. This phenomenon indicates that Bcl-3 is also essential for T-cell metabolism, and the knockout of Bcl-3 decreases metabolic activity in Jurkat cells.

Our study also revealed that the expression of the key enzyme mTOR was significantly downregulated by analyzing the changes in metabolism-related genes in Jurkat cells after Bcl-3 knockout at the protein and gene levels. mTOR mainly exists in the form of mTORC1 and mTORC2 complexes and is a key protein that regulates the metabolism of glucose, lipids and proteins [30–32]. Recently, mTOR has been identified to have an essential effect on the immune system [33, 34]. Mounting evidence suggests that it is a critical molecule for sensing the immune microenvironment and determining the function of immune cells [35–37]. Notably, mTORC1 can coordinate metabolic pathways to regulate T-cell proliferation, differentiation, and activation functions [38–41]. This raises questions as to whether the Bcl-3 molecule can control T-cell metabolism through the mTOR pathway to affect the proliferation and activation functions of T cells. More importantly, it has been reported that Raptor is a characteristic protein of the mTORC1 complex. Our experimental results showed that the knockout of Bcl-3 could significantly increase the phosphorylation of Raptor. It is well established that the Raptor-mTORC1 coordinated metabolic pathway is functionally important in regulating the differentiation and activation of T cells [42]. In this respect, it has been shown that mice lacking Raptor exhibit impaired T-cell differentiation [43]. Silencing mTOR or Raptor with shRNA in activated T cells has been found to enhance the differentiation of Tfh cells [44]. In addition, the function of Treg inhibition was significantly impaired, and a severe inflammatory reaction occurred in mice with Treg-specific deletion of Raptor [45, 46]. Therefore, whether the metabolic pathway of Bcl-3 regulation of T-cell function in this experiment involves the Raptor-mTORC1 pathway remains to be investigated, which will be the focus of our future studies. Our results may provide novel insights into the regulatory role of Bcl-3 in T-cell energy metabolism for the prevention and treatment strategies of immune diseases.

## Conclusions

This study provides novel insights and targets to further explore the specific mechanisms by which Bcl-3 regulates the mTOR pathway, maintains immune homeostasis, and prevents inflammation and autoimmune diseases by targeting Bcl-3 molecules.

### Electronic supplementary material

Below is the link to the electronic supplementary material.

Supplementary Material 1

## Data Availability

The datasets used and/or analyzed during the current study are available from the corresponding author on reasonable request.

## References

[1] Geginat J, Paroni M, Facciotti F, Gruarin P, Kastirr I, Caprioli F (2013). The CD4-centered universe of human T cell subsets. Semin Immunol.

[2] Pennock ND, White JT, Cross EW, Cheney EE, Tamburini BA, Kedl RM (2013). T cell responses: naive to memory and everything in between. Adv Physiol Educ.

[3] Gerriets VA, Rathmell JC (2012). Metabolic pathways in T cell fate and function. Trends Immunol.

[4] Geltink RIK, Kyle RL, Pearce EL (2018). Unraveling the Complex Interplay between T Cell metabolism and function. Annu Rev Immunol.

[5] Jacobs SR, Herman CE, Maciver NJ, Wofford JA, Wieman HL, Hammen JJ (2008). Glucose uptake is limiting in T cell activation and requires CD28-mediated akt-dependent and independent pathways. J Immunol.

[6] van der Windt GJ, Pearce EL (2012). Metabolic switching and fuel choice during T-cell differentiation and memory development. Immunol Rev.

[7] Palmer S, Chen YH (2008). Bcl-3, a multifaceted modulator of NF-kappaB-mediated gene transcription. Immunol Res.

[8] Kerr LD, Duckett CS, Wamsley P, Zhang Q, Chiao P, Nabel G (1992). The proto-oncogene bcl-3 encodes an I kappa B protein. Genes Dev.

[9] Carmody RJ, Ruan Q, Palmer S, Hilliard B, Chen YH (2007). Negative regulation of toll-like receptor signaling by NF-kappaB p50 ubiquitination blockade. Science.

[10] Franzoso G, Bours V, Azarenko V, Park S, Tomita-Yamaguchi M, Kanno T (1993). The oncoprotein Bcl-3 can facilitate NF-kappa B-mediated transactivation by removing inhibiting p50 homodimers from select kappa B sites. EMBO J.

[11] Annemann M, Plaza-Sirvent C, Schuster M, Katsoulis-Dimitriou K, Kliche S, Schraven B (2016). Atypical IkappaB proteins in immune cell differentiation and function. Immunol Lett.

[12] Herrington FD, Nibbs RJ. Regulation of the adaptive Immune response by the IkappaB family protein Bcl-3. Cells. 2016;5(2). 10.3390/cells5020014 PMC4931663 27023613

[13] Sun SC (2017). The non-canonical NF-kappaB pathway in immunity and inflammation. Nat Rev Immunol.

[14] Mitchell TC, Thompson BS, Trent JO, Casella CR (2002). A short domain within Bcl-3 is responsible for its lymphocyte survival activity. Ann N Y Acad Sci.

[15] Chilton PM, Mitchell TC (2006). CD8 T cells require Bcl-3 for maximal gamma interferon production upon secondary exposure to antigen. Infect Immun.

[16] Bassetti MF, White J, Kappler JW, Marrack P (2009). Transgenic Bcl-3 slows T cell proliferation. Int Immunol.

[17] Tang W, Wang H, Claudio E, Tassi I, Ha HL, Saret S (2014). The oncoprotein and transcriptional regulator Bcl-3 governs plasticity and pathogenicity of autoimmune T cells. Immunity.

[18] Corn RA, Hunter C, Liou HC, Siebenlist U, Boothby MR (2005). Opposing roles for RelB and Bcl-3 in regulation of T-box expressed in T cells, GATA-3, and th effector differentiation. J Immunol.

[19] Mufazalov IA, Kuschmann J, Andruszewski D, Masri J, Gabriel LA, Adams P (2017). Balanced Bcl-3 expression in murine CD4(+) T cells is required for generation of encephalitogenic Th17 cells. Eur J Immunol.

[20] Zhang S, Gao J, Liu S, Yu L, Zhang W, Liang Y (2021). Transcription Coactivator BCL3 Acts as a potential Regulator of lipid metabolism through the Effects on inflammation. J Inflamm Res.

[21] Tassi I, Claudio E, Wang H, Tang W, Ha HL, Saret S (2014). The NF-kappaB regulator Bcl-3 governs dendritic cell antigen presentation functions in adaptive immunity. J Immunol.

[22] Everts B, Pearce EJ (2014). Metabolic control of dendritic cell activation and function: recent advances and clinical implications. Front Immunol.

[23] Lee YS, Wollam J, Olefsky JM (2018). An Integrated View of Immunometabolism. Cell.

[24] Ganeshan K, Chawla A (2014). Metabolic regulation of immune responses. Annu Rev Immunol.

[25] Rangelova S, Kirschnek S, Strasser A, Hacker G (2008). FADD and the NF-kappaB family member Bcl-3 regulate complementary pathways to control T-cell survival and proliferation. Immunology.

[26] Mitchell TC, Hildeman D, Kedl RM, Teague TK, Schaefer BC, White J (2001). Immunological adjuvants promote activated T cell survival via induction of Bcl-3. Nat Immunol.

[27] Vander Heiden MG, Cantley LC, Thompson CB (2009). Understanding the Warburg effect: the metabolic requirements of cell proliferation. Science.

[28] Vendelbo MH, Nair KS (2011). Mitochondrial longevity pathways. Biochim Biophys Acta.

[29] Pearce EL, Pearce EJ (2013). Metabolic pathways in immune cell activation and quiescence. Immunity.

[30] Chen Y, Zhou X (2020). Research progress of mTOR inhibitors. Eur J Med Chem.

[31] El Hiani Y, Egom EE, Dong XP (2019). mTOR signalling: jack-of-all-trades (1). Biochem Cell Biol.

[32] Zou Z, Tao T, Li H, Zhu X (2020). mTOR signaling pathway and mTOR inhibitors in cancer: progress and challenges. Cell Biosci.

[33] Guertin DA, Sabatini DM (2007). Defining the role of mTOR in cancer. Cancer Cell.

[34] Laplante M, Sabatini DM (2012). mTOR signaling in growth control and disease. Cell.

[35] Powell JD (2006). The induction and maintenance of T cell anergy. Clin Immunol.

[36] Vanasek TL, Khoruts A, Zell T, Mueller DL (2001). Antagonistic roles for CTLA-4 and the mammalian target of rapamycin in the regulation of clonal anergy: enhanced cell cycle progression promotes recall antigen responsiveness. J Immunol.

[37] Delgoffe GM, Powell JD (2009). mTOR: taking cues from the immune microenvironment. Immunology.

[38] Delgoffe GM, Kole TP, Zheng Y, Zarek PE, Matthews KL, Xiao B (2009). The mTOR kinase differentially regulates effector and regulatory T cell lineage commitment. Immunity.

[39] Ohtsubo M, Theodoras AM, Schumacher J, Roberts JM, Pagano M (1995). Human cyclin E, a nuclear protein essential for the G1-to-S phase transition. Mol Cell Biol.

[40] Chen H, Zhang L, Wang P, Su H, Wang W, Chu Z (2017). mTORC2 controls Th9 polarization and allergic airway inflammation. Allergy.

[41] Liu M, Zhang J, Pinder BD, Liu Q, Wang D, Yao H et al. WAVE2 suppresses mTOR activation to maintain T cell homeostasis and prevent autoimmunity. Science. 2021;371(6536). 10.1126/science.aaz4544 33766857

[42] Yang K, Blanco DB, Chen X, Dash P, Neale G, Rosencrance C et al. Metabolic signaling directs the reciprocal lineage decisions of alphabeta and gammadelta T cells. Sci Immunol. 2018;3(25). 10.1126/sciimmunol.aas9818 PMC6230375 29980617

[43] Yang K, Shrestha S, Zeng H, Karmaus PW, Neale G, Vogel P (2013). T cell exit from quiescence and differentiation into Th2 cells depend on Raptor-mTORC1-mediated metabolic reprogramming. Immunity.

[44] Ray JP, Staron MM, Shyer JA, Ho PC, Marshall HD, Gray SM (2015). The Interleukin-2-mTORc1 kinase Axis defines the signaling, differentiation, and metabolism of T Helper 1 and follicular B helper T cells. Immunity.

[45] Zeng H, Yang K, Cloer C, Neale G, Vogel P, Chi H (2013). mTORC1 couples immune signals and metabolic programming to establish T(reg)-cell function. Nature.

[46] Sun IH, Oh MH, Zhao L, Patel CH, Arwood ML, Xu W (2018). mTOR complex 1 signaling regulates the generation and function of Central and Effector Foxp3(+) Regulatory T cells. J Immunol.

