# Supplementary figures and images for: Bcl-3 regulates T cell function through energy metabolism
Source: BMC Immunol. 2023 Oct 4;24:35. doi: 10.1186/s12865-023-00570-3 (PMC10552310; doi:10.1186/s12865-023-00570-3)

Actin


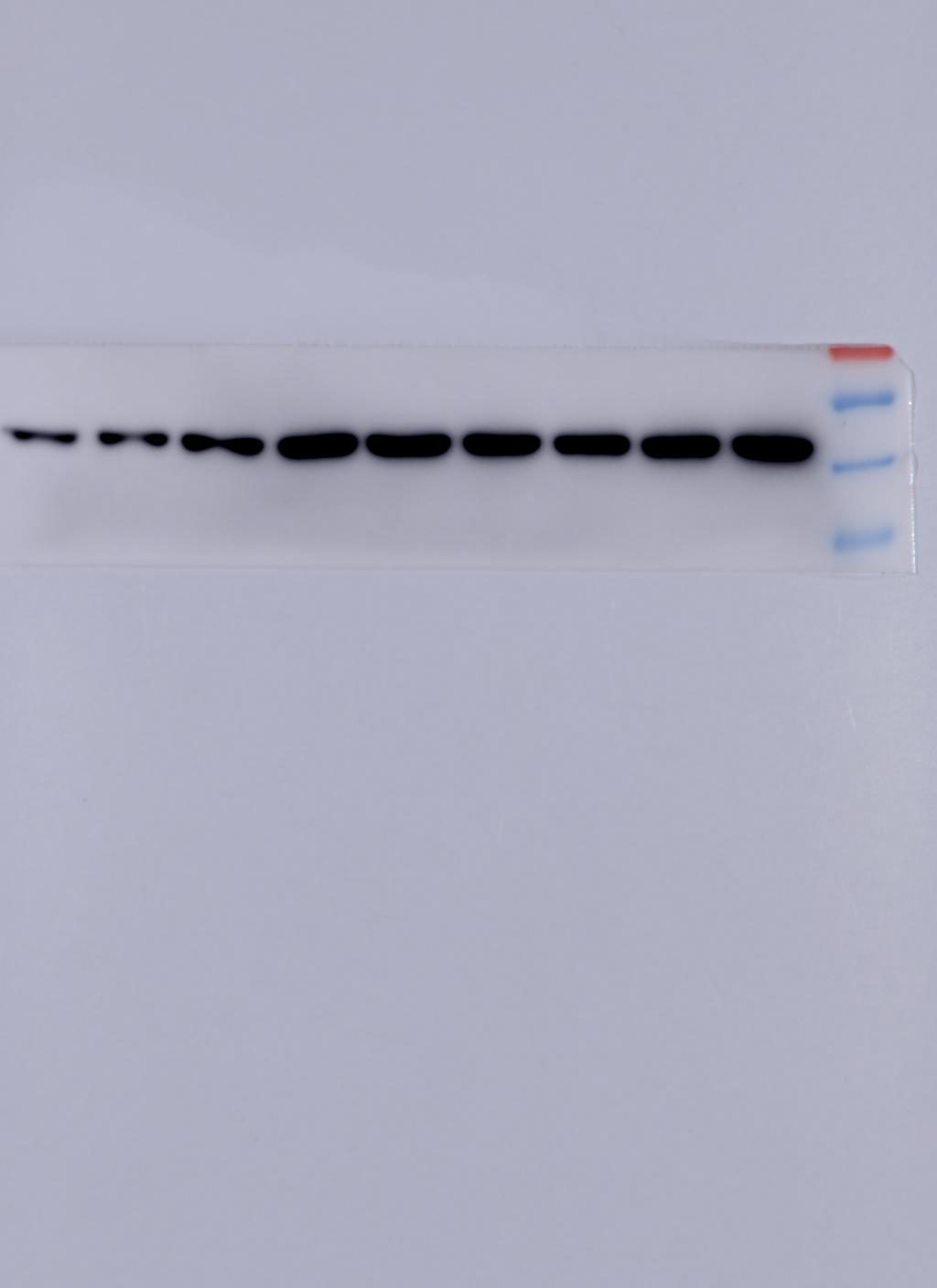


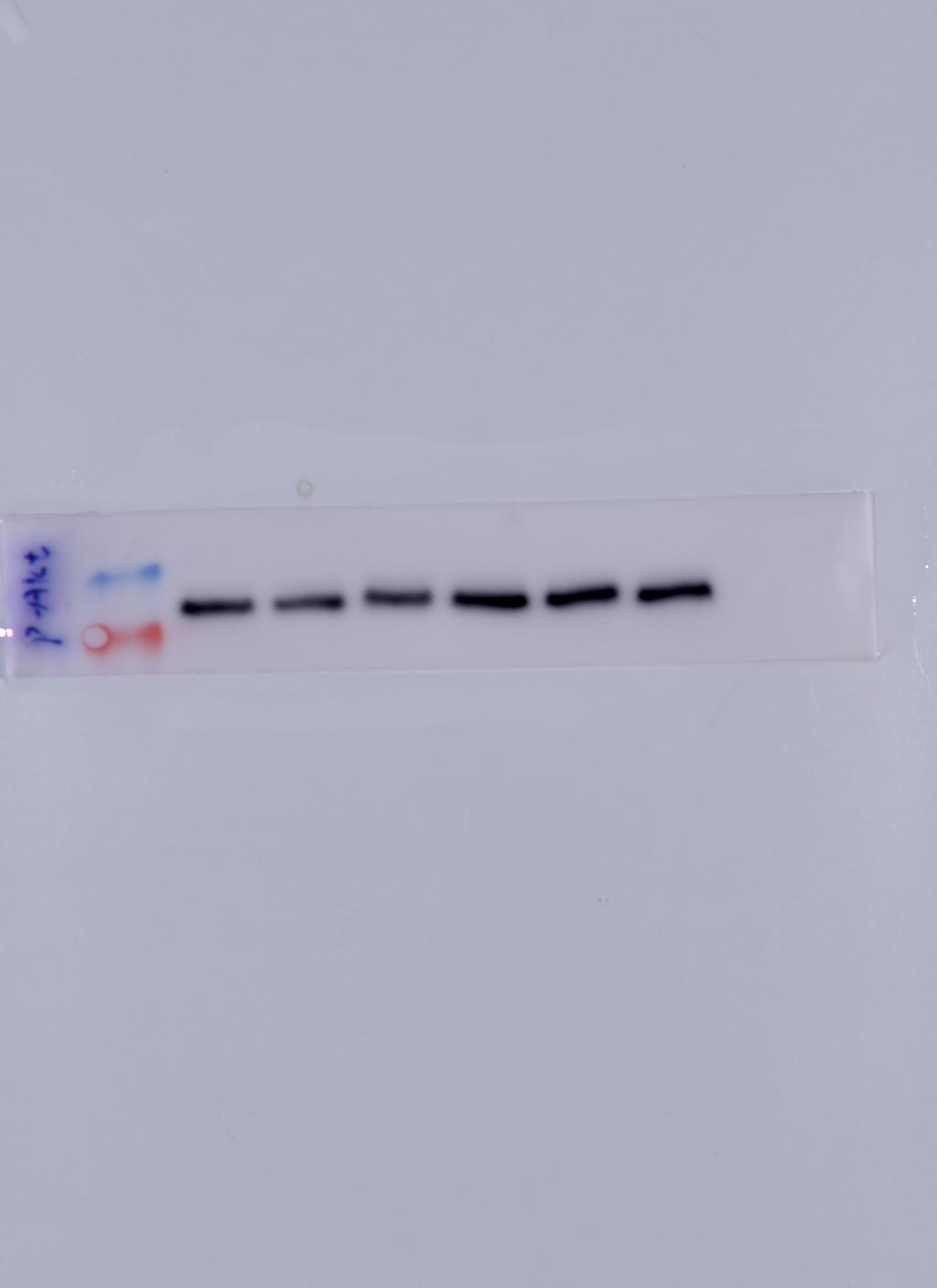


Akt


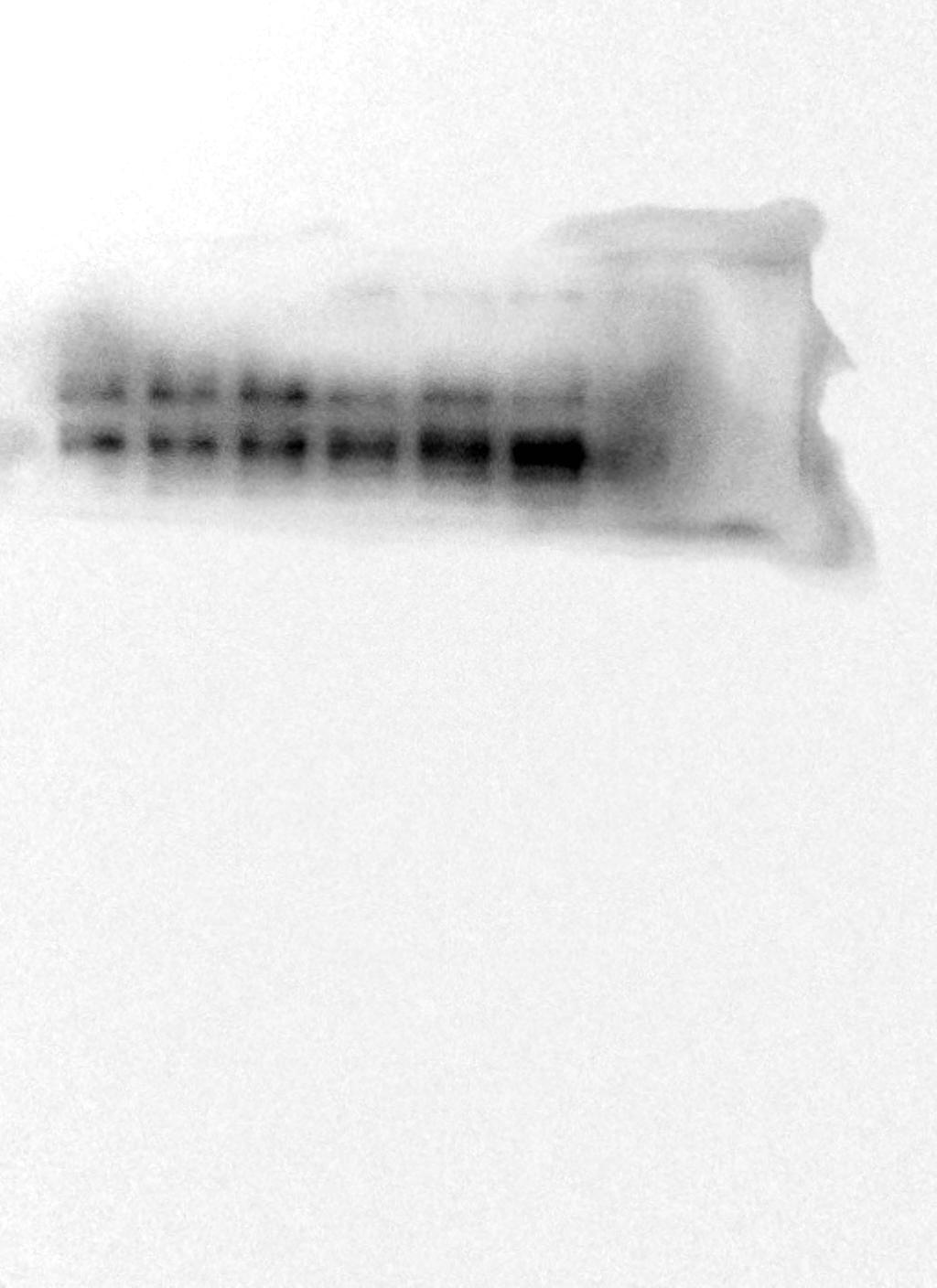
p-Akt


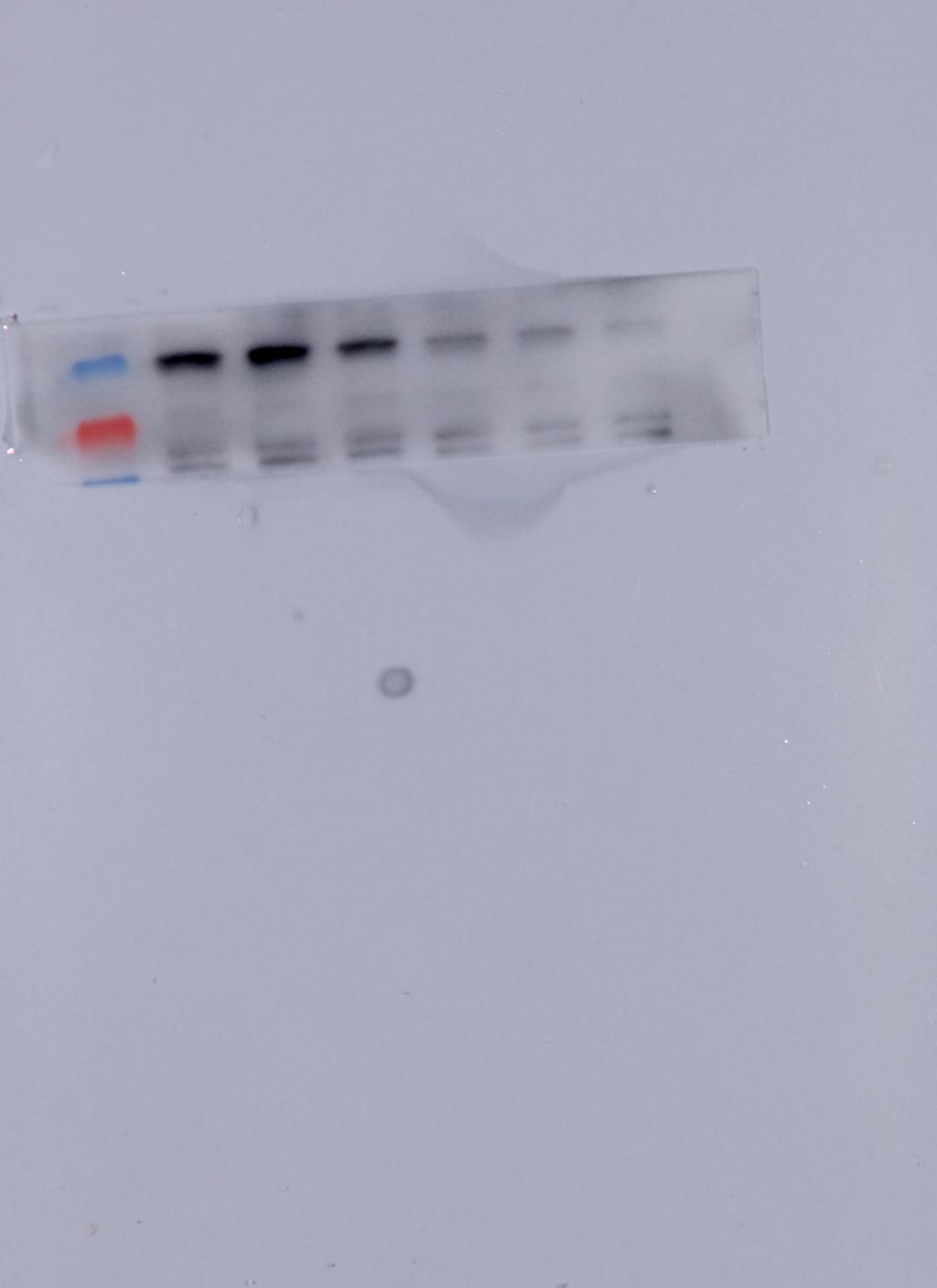
Bcl-3


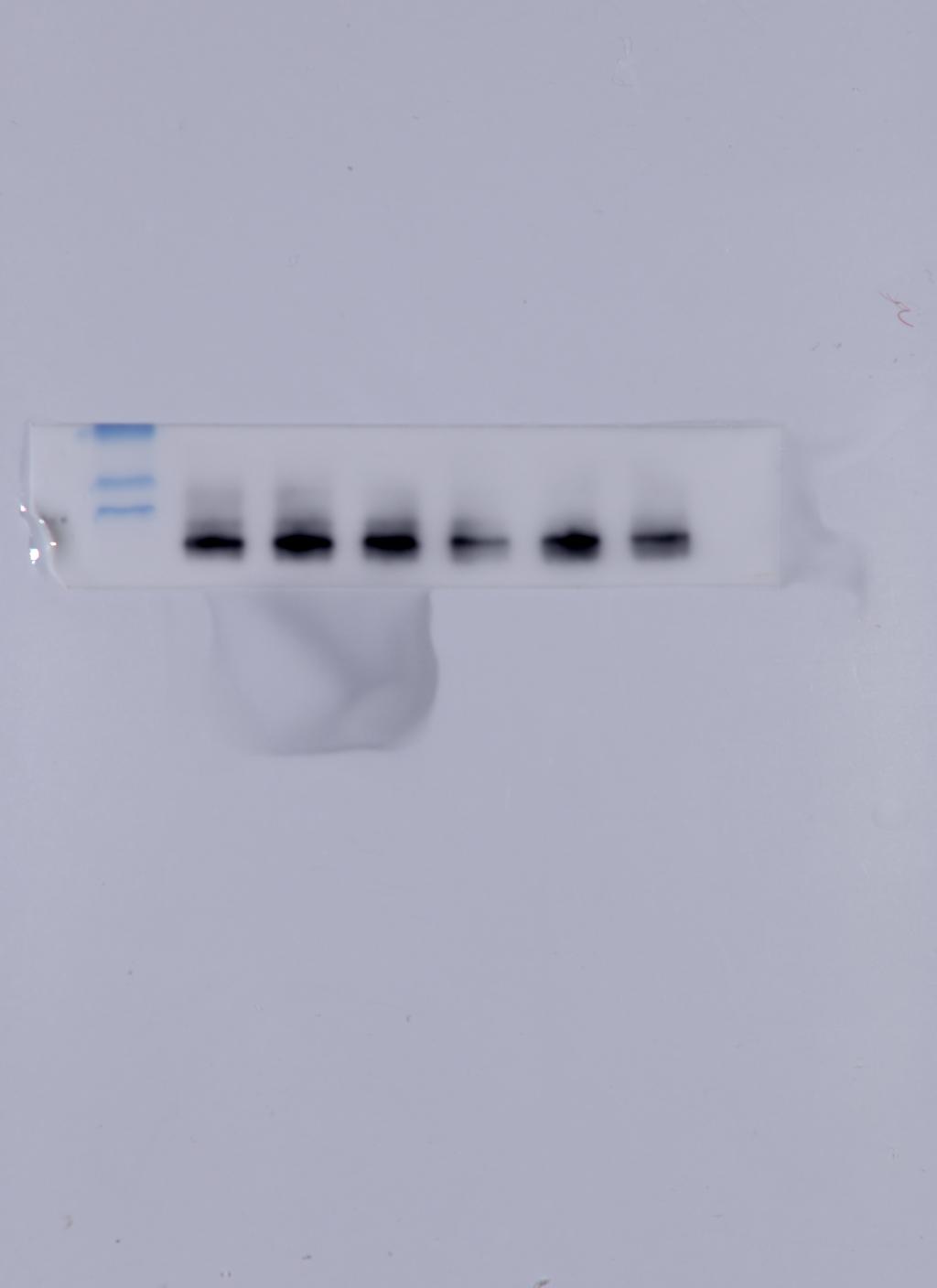
mTOR


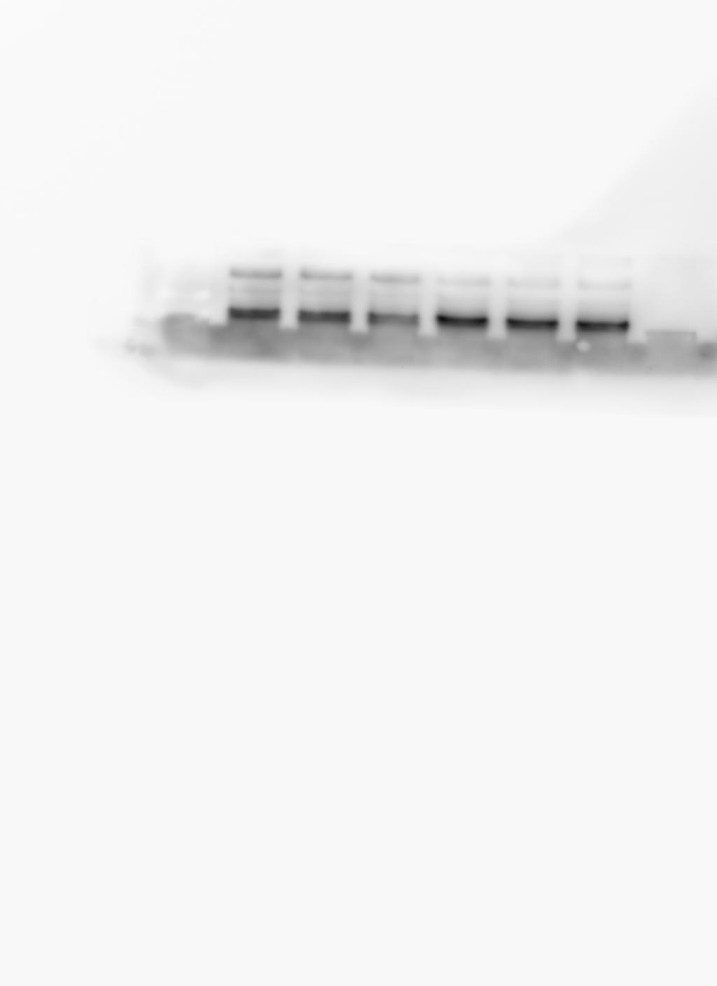
p-Raptor

Raptor


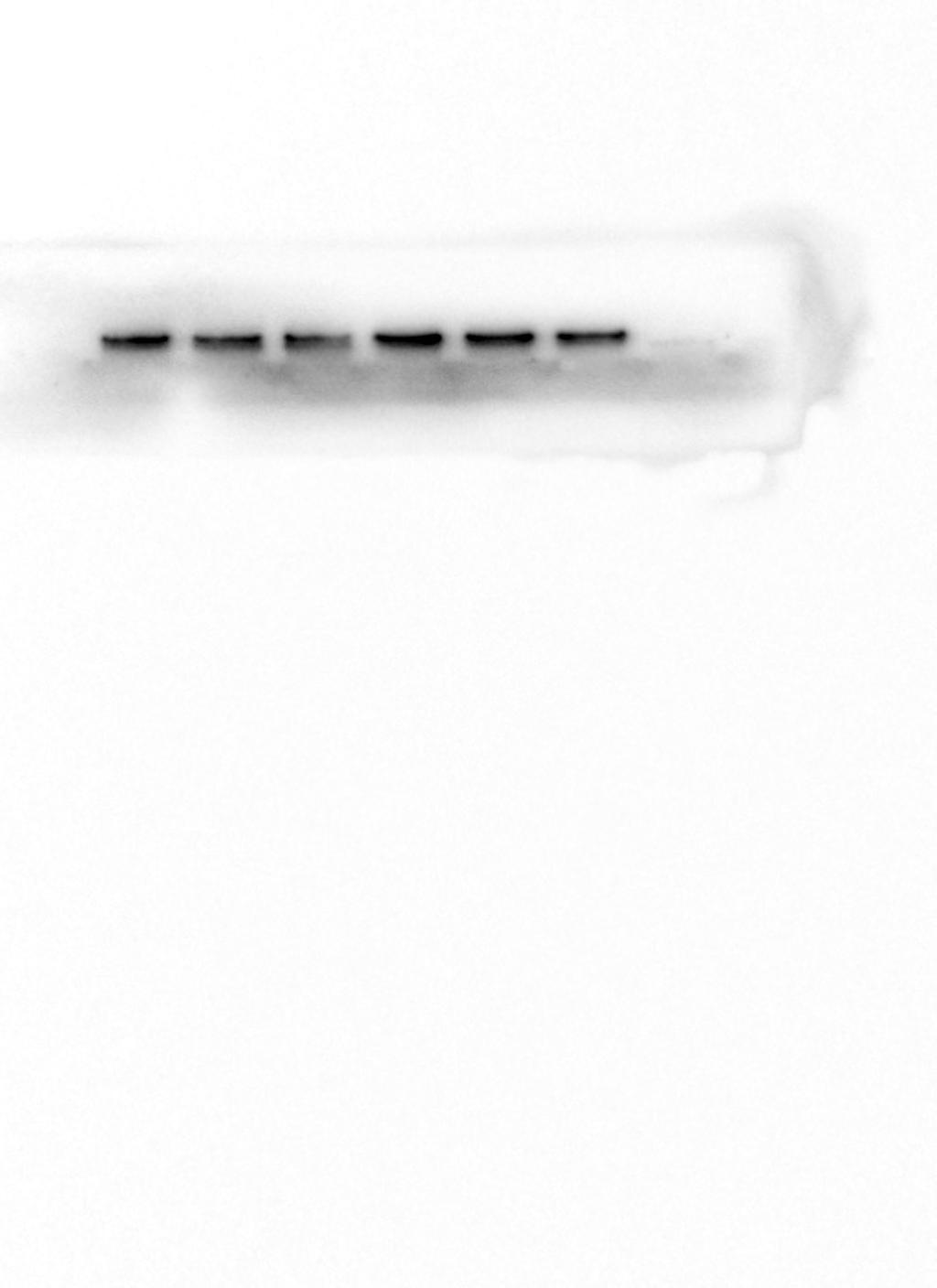


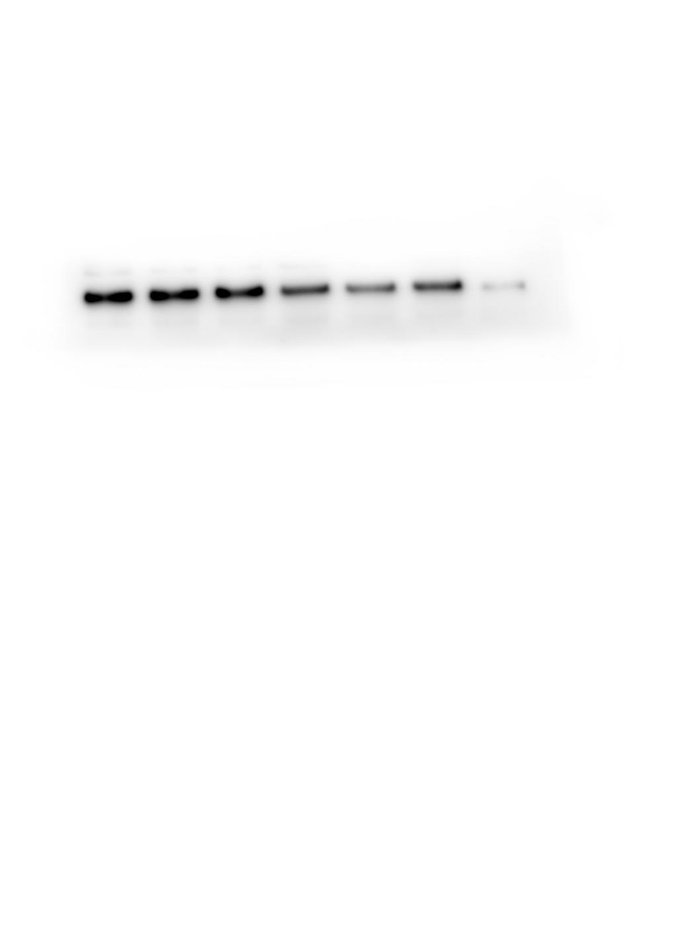
SREBP1

Supplement: Supplementary file 1 — Supplementary Material 1 [file 12865_2023_570_MOESM1_ESM.docx]
